# Use of Medicinal Plants during Pregnancy, Childbirth and Postpartum in Southern Morocco

**DOI:** 10.3390/healthcare10112327

**Published:** 2022-11-21

**Authors:** Nadia Kamel, Rachida El Boullani, Yahia Cherrah

**Affiliations:** 1 Laboratory of Pharmacology, Faculty of Medicine and Pharmacy, Mohammed V University in Rabat, Rabat 10100, Morocco; 2 Laboratory of Biotechnology and Valorization of Natural Resources, Faculty of Sciences, University Ibn Zohr, Agadir 80000, Morocco

**Keywords:** medicinal plants, pregnancy, childbirth, prevalence, associated factors, Morocco

## Abstract

Southern Morocco, particularly the Guelmim-Oued Noun region, is rich in a wide diversity of plant species. Pregnant women in this region use medicinal plants during pregnancy and childbirth for various purposes; however, the use of these plants has never been documented. The objectives of this study are threefold: to estimate the prevalence of medicinal plant uses by pregnant women in the province of Guelmim, Morocco, to describe the traditional practices of self-medication and to determine the associated factors. This is a multicenter cross-sectional study with descriptive and analytical approaches. Data were collected using an interview questionnaire, which was administered to pregnant women at health care centers and hospitals in the province of Guelmim. A total of 560 women participated in this study. The prevalence of medicinal plant use was 66.96%. *Artemisia herba-alba* Asso, *Thymus maroccanus* Ball., *Trigonella foenum-graecum* L., *Aloysia citriodora* Palau, *Lepidium sativum* L. and *Cuminum cyminum* L. were the plants with the highest UV. Pain, the induction and facilitation of childbirth, flu syndrome and anemia were the most listed reasons for use. The use of medicinal plants was significantly associated with the level of education (chi-square = 15.651; *p* = 0.004), and pregnancy monitoring (chi-square = 5.283; *p* = 0.028). In the province of Guelmim, the prevalence of the use of medicinal plants by women during pregnancy and childbirth is high. Further research is necessary in order to explore potential associated risks and complications.

## 1. Introduction

The concept of traditional Arabic herbal medicine has increasingly attracted interest among traditional herbalists and the scientific community worldwide. According to the World Health Organization (WHO), 80% of the world’s population, especially in developing countries, uses a variety of traditional medicines for their primary health care [1]. In the Arab world, traditional medicine has always been practiced despite advances in modern medicine.

In Morocco, the number of medicinal plants is estimated to be about 600 species [2,3], and more than half of them (360 species) are used for the treatment of a wide variety of diseases [4,5]. Traditional medicine is a very important form of health care for many rural populations, especially in the mountainous regions of the Atlas [6,7]. It has been estimated that 50% to 75% of the Moroccan population depends on the use of medicinal plants for their remedies [8].

Pregnancy is accompanied by physical and physiological changes in the female’s body leading to many pregnancy-related problems, including nausea, vomiting, constipation and heartburn [9]. Pregnant women tend to turn to natural medicinal plants (MPs) rather than prescription drugs to deal with these changes, especially because they are concerned about the safety of the fetus [10].

The use of MPs to treat maternal complications has been observed in many populations [11,12,13], and showed a wide range of prevalence (7% to 55%) based on the geographical area and the socio-cultural and ethnic aspect of the group investigated [14,15]. It has been shown that more than half of pregnant women in Alexandria city, Egypt, reported the effectiveness of herbal medicines to relieve ailments during pregnancy [16]. This usage rate is even higher in places where herbal medicine is a reference therapy, such as in Ivory Coast where 90.3% of pregnant women use MPs during pregnancy. However, this practice is ignored by midwives during antenatal visits [17].

Previous studies have documented that the most commonly used MPs by pregnant women are: anise, fenugreek, ginger, cranberry, chamomile, licorice, fennel, aloe, valerian, Echinacea, almond, oil, propolis and castor oil [10,14,16]. Medicinal plants are preferred over prescription drugs due to the belief that traditional medicine is safer than modern medicine. Pregnant women prefer the use of medicinal plants rather than prescription drugs, even though information on their safety and efficacy are very limited. In addition, the side effects associated with the use of MPs are sometimes accepted by users [14,18,19].

The use of medicinal plants and supplements by pregnant women may have unclear effects during pregnancy or serious complications on the fetus [20,21]. Risks and long-term negative effects on the health of the mother and the fetus could be affected by herbal medicine, such as maternal morbidity, mortality or neonatal morbidity, tumors, inflammation and gastrointestinal diseases [22,23]. Pregnant women have reported side effects after drinking herbal tea containing a mixture of herbs (constipation) or after topical application of aloe or almond oil (rashes and itching) [14]. Several authors have described the potential adverse effects of certain herbal medicines during pregnancy, such as the side effects associated with the use of fenugreek (*Trigonella foenum-graecum*), harmel (*Peganum harmala* L.), nigella (*Nigella sativa* L.), Rosemary (*Rosmarinus officinalis* L.) and many others [24,25,26].

Moroccan women resort to either traditional medicine or modern medicine to treat problems related to pregnancy based on local habits and ancestral beliefs. However, data on the extent of the use of MPs and MP-based products during pregnancy are limited [27]. It has been documented that 60% of women in the city of Marrakech, Morocco, use medicinal plants during pregnancy and maternity for various purposes [28]. Women in the Guelmim-Oued Noun region, which is known for its richness in medicinal plant species [29], are known to use medicinal plants during pregnancy and childbirth. However, the use of these plants has never been documented. The aim of this study was to document the use of medicinal plants by pregnant and postpartum women in the Guelmim-Oued Noun region in Morocco and identify the associated factors.

## 2. Materials and Methods

### 2.1. The Study Area

**Figure 1:**
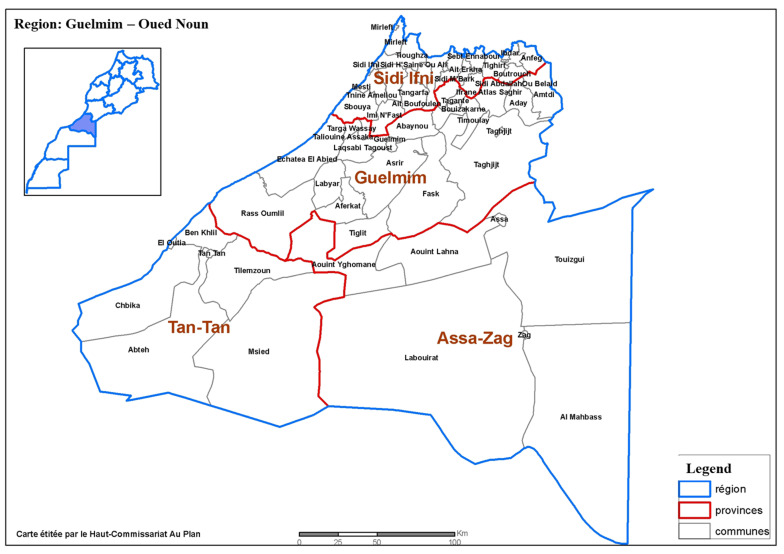
Map of the province of Guelmim and boundaries of the study area; High commission for planning–Guelmim regional directorate. (https://www.hcp.ma/region-guelmim/Presentation-de-la-region_a1.html (accessed on 1 April 2021)).

The Province of Guelmim is part of the Gulemim-Oued Noun region; it covers an area of 10,783 Km^2^, which account for 18% of the territory of the region (Figure 1). It is bordered to the north by the provinces of Tiznit and Sidi Ifni, to the south by the provinces of Tan-Tan and Assa-Zag, to the east by the provinces of Tata and Assa-Zag and to the west by the Atlantic Ocean. Administratively, the province of Guelmim is made up of two territories and 20 municipalities, 2 of which are urban. The province of Guelmim constitutes a buffer zone between the Moroccan Sahara and the Souss plain. The proximity of the Atlantic Ocean attenuates the effects of the Sahara Desert close to the ocean. The climate is marked by a variability in precipitation. The average annual rainfall varies between 90 and 120 mm. The maximum and minimum temperatures are 45 °C and 0.1 °C, respectively. The average annual temperature is around 20.5 °C. The winds are very frequent causing sand accumulations of different forms.

### 2.2. Type of Study

This is a cross-sectional, descriptive and analytical study conducted in the province of Gulemim.

### 2.3. Study Population

The study opted for a comprehensive sampling by including all the pregnant women who presented themselves for the prenatal consultation (PNC) at the level of all structures within the primary health care facilities network in the province of Guelmim, namely: 05 s-level rural health centers with a delivery unit (127 pregnant women monitored), 04 first-level rural health centers (28 pregnant women monitored) and 09 first-level urban health centers (305 pregnant women monitored). For women who had given birth, the study exhaustively recruited all women who admitted themselves to the hospital maternity ward during the study period in order to collect as much information as possible on the therapeutic uses of the medicinal plants used during pregnancy and childbirth.

Inclusion criteria: All pregnant women who presented themselves for prenatal consultation at all structures of the network of primary health care facilities and women admitted for childbirth at the maternity hospital in the province of Guelmim.

Exclusion criteria: Women who refused to participate in the study.

### 2.4. Questionnaire

A researcher-administered questionnaire was used for data collection. The first part of the questionnaire was devoted to the socio-demographic characteristics of the women surveyed (age, level of education, marital status, language spoken, height, weight, place of residence, professional occupation and income). The second part included questions related to pregnancy (reason for consultation, parity, gestation, pregnancy monitoring, medical, surgical and gynecological-obstetrics history, pregnancy at risk, type of pregnancy at risk by using prenatal consultation follow-up sheets as part of the pregnancy and childbirth follow-up program (PCFP)). The last part of the questionnaire covered information related to the use of MPs (vernacular name of each species, mode of preparation and administration, period of use and reasons for use).

First, a list of the vernacular names of the medicinal plants used by the respondents was prepared by referring to the taxonomy of Fennane et al. (1999, 2007 and 2014) on the flora of Morocco [30,31,32]. The scientific names of the plant species were determined based on the list of plants presented on the site (http://www.theplantlist.org (accessed on 1 May 2021). Validation of the concordance between the vernacular names, the botanical names and the names in French were carried out at the Laboratory of Biotechnology and Valorization of Natural Resources of the Faculty of Sciences, University Ibn Zohr, Agadir, Morocco.

### 2.5. Ethical Considerations

The present study was approved by the ethics committee for biomedical research at the Faculty of Medicine and Pharmacy of Rabat, Morocco, under the number 29/19. The consent for participation in the study was obtained before each interview by proving to the participants all the information related to the nature of the study and its objective. The women included in the study were identified by an anonymous study number corresponding to each participant. In addition, the confidentiality of the data collected was rigorously respected.

### 2.6. Statistical Analysis

Quantitative variables were presented as the median ± interquartile range. Qualitative variables were described using frequencies and percentages. A test of association between categorical variables was carried out using chi-square test or Fischer’s exact test in the case where the conditions of the chi-square test were not met. The significance level was set at 5%. The data collected were coded, entered, processed and analyzed using SPSS version 24.0 software.

Ethnobotanical data were analyzed using the use value (UV) and relative frequency citation (RFC) to determine which species were well known and most used by the women in this study. UV is a quantitative index that demonstrates the relative importance of locally known species [33,34].

RFC shows the local importance of each species and is obtained by dividing the number of informants mentioning a useful species (frequency citation (FC)) by the total number of informants in the survey (N) [35]. This index was calculated using the following formula: RFC = FC/N (0 < RFC < 1).

Use value (UV) was calculated according to Phillips and Gentry et al. 1994 [33] using the following formula:

UV = ∑/N

where “∑ “refers to the number of uses mentioned by the informants for a given species and “N” refers to the total number of informants interviewed. If a plant secures a high UV score, that indicates that there are many use reports for that plant, while a low score indicates fewer use reports cited by the respondents.

## 3. Results

### 3.1. General Characteristics of the Population Surveyed

A total of 560 pregnant and postpartum women were interviewed, 305 pregnant women were interviewed at the first-level urban health centers, 127 pregnant women were interviewed at the second-level rural health centers with delivery unit, 28 pregnant women were interviewed at the first-level rural health centers and 100 women delivered their interviews at the maternity hospital (Table 1).

The characteristics of the population surveyed are presented in Table 2. The median age of the women surveyed was 30 years with an interquartile range (IQR) of 10.14 years. Almost all (98.9%) of the respondents were married. Women with no level of education represented 24.5% of the respondents, and those with a university level represented 13.1%. Women from urban areas made up 66% of the respondents. Women covered by health insurance represented 23.8%, and those covered by beneficiaries of the medical assistance scheme for the economically underprivileged persons (RAMED) represented 50.5%. Half of the participants had very low income, while 10.6% were rich. As for the occupation of the husbands, 96.6% of the spouses worked intermittently. Women with previous gynecological-obstetrics, medical and surgical history represented 27%, 19% and 6%, respectively. In terms of gestation, 29% were first-timers and 27% second-timers. Nulliparous women represented 11% of the respondents, second parents 26.5% and third parents 17.7% Pregnant women under medical control (pregnancy follow-up) represented 93.7% of the respondents, and 34.3% of them were diagnosed with high-risk pregnancies.

### 3.2. Characteristics of the Women Using MPs

Most of the women interviewed had no medical-surgical or gynecological-obstetrical history; 31.8% were primiparous, 26.2% second gesture, 23.3% third gesture, 18.9% ≥ fourth gesture and 28.2% primiparous, 26.2% second parity, 18.7% third parity and 15.3% were multiparous (>4). Among the participating women who were followed prenatally (95.1%), 32.8% were diagnosed with high-risk pregnancies. Specifically, 45.03% were diagnosed with anemia, 18.54% were diagnosed with gestational diabetes, 14.57% with hypertension and 3.97% with pre-eclampsia (Table 2).

### 3.3. Prevalence and Risk Factors Linked to the Use of MPs

**Figure 2:**
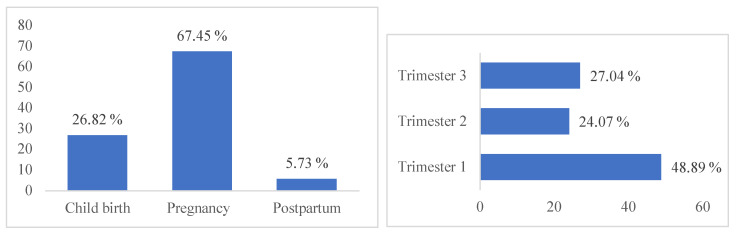
Prevalence of MP usage during pregnancy (Trimester 1, 2 and 3) at childbirth and at postpartum.

This study showed that 67.45% of the respondents used MPs during pregnancy, 26.82% during childbirth and 5.73% at postpartum (Figure 2). With regard to the period during which MPs were used, 48.89% of women used MPs during the first trimester, 24.07% in the second trimester and 27.04% in the third trimester (Figure 2). In addition, data analysis showed that the use of MPs by pregnant women is related to the level of education (*p* = 0.004) and also with the pregnancy follow-up (*p* = 0.028) (Table 2).

### 3.4. Monograph of Medicinal Plants Used by Pregnant Women and Reasons for Use

A list of the plants identified during this study is presented in Table 3. They are presented according to families, scientific names and vernacular names, the modes of preparation, the use value (UV) and the relative frequency of citation (RFC). Data analysis revealed the presence of 43 different MPs used by pregnant women. They belong to 23 botanical families, the most represented ones were Apiaceae (seven species), Lamiaceae (five species), Asteraceae and Fabaceae (four species), while other families were represented by either one or two species.

The use value (UV) of the species (Table 3) enabled us to identify six species with high UVs; they were: *Artemisia herba-alba* Asso (UV = 0.059), *Thymus maroccanus* Ball. (UV = 0.045), *Trigonella foenum-graecum* L. and *Aloysia citriodora* Palau (UV = 0.037), *Lepidium sativum* L. (UV = 0.035) and *Cuminum cyminum* L. (UV = 0.032).

The FRC index (Table 3) indicated that eight medicinal plants are used at high level, either alone or in combination with other plants. Among them, four species have the highest RFC value (higher than 0.080); they were: *A. herba-alba* Asso (RFC = 0.195), *T. maroccanus* Ball. (RFC = 0.153), *A. citriodora* Palau (FRC = 0.097) and *T. foenum-graecum* L. (FRC = 0.081). Pregnant women used different MPs depending on the stage of pregnancy (Table 3):

- First trimester of pregnancy: white mugwort, thyme and fenugreek.
- Second trimester: thyme, white mugwort, olive tree and sesame.
- Third trimester: white mugwort, thyme and fenugreek.
- Labor and delivery: white mugwort, thyme, garden cress and saffron.
- Postpartum: garden cress, fenugreek and white mugwort.

**Figure 3:**
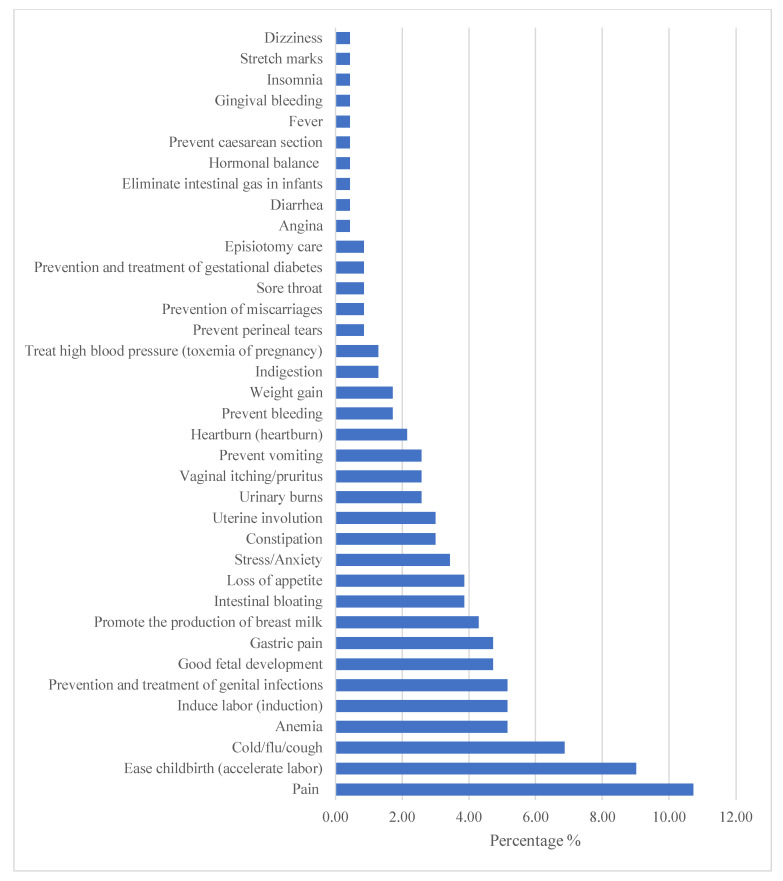
Reasons for the use of medicinal plants by women in Guelmim.

These MPs were used for different purposes such as: pain, the facilitation of childbirth, against flu syndrome, anemia and the induction of labor (Figure 3).

### 3.5. Mode of Preparation and Route of Administration

The present study revealed that various routes are used for the administration of herbal preparations. The oral route was the predominant one (73.21%), followed by vaginal (20.53%) and then the nasal route (3.84%) (Table 4). With regard to the mode of preparation, decoction was the preferred mode (34.86%) followed by infusion (31.27%) (Table 5).

### 3.6. Source of Information

This survey showed that 46% of the pregnant women interviewed refer to family members for information related to MP use, while 34% refer to neighbors and friends (Table 6).

## 4. Discussion

This is the first study on traditional self-medication practices related to pregnancy and childbirth in the province of Guelmim, Morocco. The aim of this study was to estimate the prevalence of the use of medicinal plants by pregnant women and to determine the associated factors. Many reports have documented the use of herbs by pregnant women for medical treatments [28,36,37,38,39,40]. In this study, we show that pregnant women in the province of Guelmim also use MPs during pregnancy. The prevalence of medical plant use differs from one country to another [28,39,41,42,43,44,45,46]. In the province of Guelmim, 66.96% of women use herbal medicine during pregnancy. This rate is significantly high compared with other similar studies conducted in other countries, such as Kenya, India, Oman, Palestine, Egypt and Taiwan [16,36,38,47,48]. These variations in prevalence could be associated with differences in the study design and/or sample dynamics [49], and also to the existence and enforcement of laws governing the marketing of medicinal plants, which also varies between countries [42,50]. The difference in socio-demographic and cultural factors also play a role in the number of women who use MPs [49]; many studies have revealed a strong belief among women in the safety of MPs during pregnancy [51,52,53], although little scientific evidence exists on their safety [50,54,55].

In a recent survey carried out in Brazil, 60% of the women who participated in the study did not believe in the existence of toxic effects of MPs, and around 39% were unaware of the potential adverse effects of MPs [56]. In addition, pregnant women tend to turn to MPs to ease complications associated with pregnancy because many medical prescriptions are contradicted by pregnant women [57].

In this study, women used herbal medicine especially during the first trimester and the labor period. This can be explained by the increase in pregnancy-related problems during these periods [58]. Herbal medicine use during the first trimester has been reported previously [40,43,55,58,59,60,61,62]. Other studies have reported that the use of medicinal plants is more common in the second trimester [63], third trimester [64] or throughout pregnancy [49].

The timing of MP usage depends on the reasons behind using herbs and varies geographically from one region to another [19,43,55,58,65,66]. This study showed that the most common reasons for using MPs by pregnant women were pain, the facilitation of childbirth, flu syndrome, anemia and the induction of labor. These results are in accordance with previous studies [17,40,61,63,64,67,68]. According to El Hajj and Holst (2020), medicinal plants can sometimes be used in the context of maternal care to treat pregnancy-related problems and often to improve the well-being of the mother and/or the unborn child [69]. For instance, ginger has been used for nausea and vomiting in the first trimester of pregnancy [37,55] and peppermint, thyme, chamomile and green tea for bloating, upset stomach and maintaining health during pregnancy [42,55,70]. Other studies have reported various other reasons for using MPs during pregnancy, such as improving the beauty and health of the fetus, and even the intelligence of the future child [16,20,49,58,59,62,71,72], enhancing fetal growth [17,68,73], as nutritional supplements, to treat skin problems and urinary tract infections [74] and to increase milk production during lactation [63]. It has also been show that MPs can ease pregnancy, improve the course of pregnancy [63], prevent/treat malaria and prevent miscarriages [17,40,73,75]. Medicinal plants are also used to fight against sleep disorders, anxiety and fatigue, to control blood sugar and cholesterol levels [76,77], maintain pregnancy, induce labor and facilitate childbirth and delivery, and for postpartum hemostasis [67].

The socio-demographic characteristics of the respondents differ from one country to another [53,60,78,79]. In this study, the median age of women who use medicinal plants was 30 years. Similar results were reported in other studies [74,80].

The present study showed that the use of medicinal plants is related to the level of education (*p* = 0.004) and to the follow-up of pregnancy (*p* = 0.028). Women with a high-level school of education make less use of MPs with a proportion of only 14%. Similar results were reported in previous studies [63,81].

The level of education is an important factor in terms of reproduction and health; the higher the level of education of women, the more it contributes to and facilitates their access to information and allows them to consult and be followed by medical specialists and to respect their instructions [82]. Illiteracy has been shown to be an important determinant associated with the use of herbal medicines [28]. In a review of 50 studies published by [83], it was reported that the use of MPs during pregnancy was significantly (*p* < 0.05) higher among women with a low level of education, higher age, married status, low socio-economic status, a low level of education of the spouse and a previous history of MP use during previous pregnancies. Other studies have revealed statistically significant differences according to age, place of residence and education of husbands, marital status, multiparity/nulliparity and many other variables [10,14,18,28,37,49,80]. This study has limitations such as not taking into consideration the psychological factors related to the use of MPs by pregnant women during pregnancy and childbirth.

The plants listed in this study have a very important place in traditional herbal medicine in Morocco, in Mediterranean countries and in the Middle East [28,40,84,85,86,87,88], indicating the therapeutic importance of these plant species in the cultural heritage of populations, their abundance and their ease of acquisition. Indeed, the south of Morocco, in particular the Guelmim-Oued Noun region, is known to have a great diversity of plant species [29]. In this study, we found that the most common plants used were *A. herba-alba* Asso, *T. maroccanus* Ball., *A. citriodora* Palau and *T. foenum-graecum* L. In other parts of Africa, the four species of MPs used mostly by pregnant women are *Zingiber officinale* (ginger), *Allium sativum* L. (garlic), *Cucurbita pepo* L. (pumpkin) and *Ricinus communis* L. (castor oil) [83], while in the Middle East, peppermint, ginger, thyme, chamomile, sage, anise, fenugreek and green tea were among the most common herbs used during pregnancy [40]. At the international level, ginger, cranberry, valerian and raspberry were among the most used plants [43]. The choice of plants is related to the culture and the season [49].

During this survey, the highest UVs were attributed to the following MPs:

- *A. herba-alba* Asso (UV = 0.059); it is one of the most used plants in the Mediterranean region to treat various diseases including diabetes, hypertension, spasmodic dysphonia and certain bacterial infections [89]. In this study, *A. herba-alba* was cited for the treatment of gestational diabetes, hypertension, problems of the digestive tract, certain genital infections and to facilitate childbirth. It has been reported that the aqueous extract of *A. herba-alba* has hypoglycemic properties [90], antihypertensive activity [91] and antimicrobial and antifungal activities [92]. In addition, a limited number of scientific studies have demonstrated the harmful effect of this plant on pregnancy and the development of the fetus and infant. A study by Laadraoui et al. (2018) [89] highlighted that transplacental exposure of A. *herba-alba* affects reproduction by increasing infertility, delayed memory function and neuromotor reflex in mouse offspring.
- *T. maroccanus* Ball. (UV = 0.045); it is a perennial aromatic shrub widely used to treat digestive, respiratory and nervous system diseases, rheumatism, bronchitis, fever, cough, wounds and many infections [93,94,95,96,97,98,99]. Pregnant women in the region of Guelmim use *T. maroccanus* Ball to treat problems related to pregnancy, namely, digestive disorders (constipation, vomiting, indigestion, etc.), genital infections, coughs, colds, the induction and acceleration of labor and also for good development of the fetus. The antiviral and analgesic activities of *T. maroccanus* oil have been documented previously [100]. In addition, a study by Belaqziz et al. (2013) [101] showed that the essential oil of *T. maroccanus* possesses antibacterial potential.
- *T. foenum-graecum* L. (UV = 0.037); it is used by women in the Gulemim region to treat anemia, facilitate childbirth, promote the production of breast milk, induce labor and prevent and treat genital infections. According to Ulbricht et al. (2008), this herb has been used to treat a range of ailments ranging from labor induction to digestion to cough [102]. Additionally, previous studies have shown that fenugreek seeds increase milk production in lactating women [103]. According to Orief et al. (2014), fenugreek should be consumed with caution during pregnancy as the seeds have the ability to lower blood sugar levels and stimulate uterine contractions [16]. According to Vu (2019), fenugreek is well tolerated without serious side effects. However, it was reported by the same authors that when fenugreek is taken with certain pharmaceutical drugs, it can exacerbate the effect of the drugs [104].

The modes of preparation of MPs, as well as the dosage, are extremely important. Pregnant women and women who have gone through childbirth in the province of Guelmim prepare MPs by different methods, especially decoction and infusion. This is consistent with other studies conducted in Morocco and elsewhere in the world [28,105,106,107]. While in other studies, medicinal plants were consumed in raw form [108], in the form of maceration [109] or even pressed and chewed [67].

In this study, the most common route of herbal administration among pregnant women was oral (73.21%). Similar findings were reported in other studies [67,83,107].

However, in other places, such as in the Ivory-cost, only 28.7% of women surveyed reported taking MPs orally [17].

With regard to the source of information or recommendation for the use of medicinal plants, family recommendation was the main source (46%), followed by recommendations from experienced people in the entourage (34%). This is in accordance with previous studies [10,16,17,40,80,83,110,111,112]. In other studies, it had been reported that 80% to 90% of the pregnant women surveyed received their information on the use of MPs from people other than health care providers [58,74,112]. However, in other places such as Russia, physician recommendations were most often cited [43].

## 5. Conclusions

The prevalence of the use of medicinal plants during pregnancy and childbirth seems high in the province of Guelmim; the level of education is one of the important determinants associated with it. The use of herbs by women must be taken into consideration during prenatal consultations in order to offer an integrated prenatal follow-up and avoid any possible complications and risks for the mother or the fetus. The results of this investigation could serve as a basis for the design and development of strategies, education and awareness programs focused on the safer use of medicinal plants that are intended, more particularly, for pregnant women and women who have given birth with a low level of education. Moreover, in-depth research seems necessary on the effects and risks associated with the use of plants during pregnancy and childbirth.

## Figures and Tables

**Table 1:** Distribution of pregnant women interviewed by health structures.

Health Structures	Number of Pregnant Women Interviewed
First-level urban health centers	305
Second-level rural health centers with delivery unit	127
First-level rural health centers	28
Maternity hospital	100
Total	560

**Table 2:** Socio-demographic characteristics of pregnant and postpartum women who participated in the study.

Variables	Total (560)	Use of Medicinal Plants		*p*-Value
		No (185)	Yes (375)	
	N (%)	N (%)	N (%)	*p* = 0.141
**Family situation**				
**Divorced**	6 (1.1)	0	6 (1.5)	
**Married**	554 (98.9)	148 (100)	406 (98.5)	
**Education level**				*p* = 0.004
**None**	135 (24.5)	52 (36.1)	83 (20.4)	
**Primary**	127 (23.0)	33 (22.9)	94 (23.1)	
**Middle school**	136 (24.7)	28 (19.4)	108 (26.5)	
**High school**	81 (14.7)	16 (11.1)	65 (16)	
**College**	72 (13.1)	15 (10.4)	57 (14.0)	
**Age (Median; Q1–Q3)**	30 years old (25–34.15)	30.01 years old (25–35)	29.43 years old (25–34)	*p* = 0.777
**Height (Median; Q1–Q3)**	161.47 cm (1.574–1.657)	161.02 cm (155.02–165.00)	161.63 cm (158.00–166.0)	*p* = 0.057
**Weight (Median; Q1–Q3)**	72.00 Kg (60.91–81.02)	72.20 Kg (61.19–82.27)	72.00 Kg (60.82–81.00)	*p* = 0.969
**Environment**				*p* = 0.971
**Nomad**	8 (1.4)	2 (1.4)	6 (1.5)	
**Rural**	182 (32.6)	49 (33.3)	133 (32.3)	
**Urban**	369 (66.0)	96 (65.3)	273 (66.3)	
**Language**				*p* = 0.669
**Amazigh**	125 (22.5)	32 (21.8)	93 (22.8)	
**Dialectal Arabic**	430 (77.5)	115 (78.2)	315 (77.2)	
**Employment of the woman**				
**Employed**	54 (9.75)	16 (10.60)	38 (9.43)	*p* = 0.680
**Unemployed**	500 (90.25)	135 (89.40)	365 (90.57)	
**Employment of the husband**				*p* = 0.117
**Employed**	533 (96.6)	137 (94.5)	396 (97.3)	
**Unemployed**	19 (3.4)	8 (5.5)	11 (2.7)	
**Income level**				*p* = 0.065
**Poor**	280 (50.1)	83 (56.5)	197 (47.8)	
**Middle class**	220 (39.4)	46 (31.3)	174 (42.2)	
**Rich**	59 (10.6)	18 (12.2)	41 (10.0)	
**Medical insurance**				*p* = 0.056
**None**	106 (19.4)	38 (26.4)	68 (16.9)	
**RAMED ^1^**	276 (50.5)	71 (49.3)	205 (50.9)	
**CNOPS**	111 (20.3)	27 (18.8)	84 (20.8)	
**CNSS**	19 (3.5)	2 (1.4)	17 (4.2)	
**Private insurance**	35 (6.4)	6 (4.2)	29 (7.2)	
**Medical history**				*p* = 0.271
**No**	454 (81.2)	124 (84.4)	330 (80.1)	
**Yes**	105 (18.8)	23 (15.6)	82 (19.9)	
**Surgical history**				*p* = 0.590
**No**	526 (94.1)	137 (93.2)	389 (94.4)	
**Yes**	33 (5.9)	10 (6.8)	23 (5.6)	
**Gynecological history**				*p* = 0.489
**No**	410 (73.3)	111 (75.5)	299 (72.6)	
**Yes**	149 (26.7)	36 (24.5)	113 (27.4)	
**Gestation**				*p* = 0.839
**1st trimester**	184 (33)	53 (35.11)	131 (31.8)	
**2nd trimester**	144 (25.8)	36 (24.5)	108 (26.2)	
**3rd trimester**	127 (22.7)	31 (21.1)	96 (23.3)	
**≥4th trimester**	104 (18.6)	27 (18.4)	77 (18.7)	
**Parity**				*p* = 0.190
**Nulliparity**	64 (11.4)	17 (11.6)	47 (11.4)	
**1st parity**	161 (28.8)	45 (30.6)	116 (28.2)	
**2nd parity**	148 (26.5)	40 (27.2)	108 (26.2)	
**3rd parity**	99 (17.7)	21 (14.3)	78 (18.9)	
**Multiparity > 4**	87 (15.6)	24 (16.3)	63 (15.3)	
**Pregnancy follow-up**				*p* = 0.028
**No**	35 (6.3)	15 (10.2)	20 (4.9)	
**Yes**	524 (93.7)	132 (89.8)	392 (95.1)	
**Assessment/ultrasound**				*p* = 0.653
**No**	65 (11.6)	15 (10.2)	50 (12.1)	
**Yes**	494 (88.4)	132 (89.8)	362 (87.9)	
**Pregnancy at risk**				*p* = 0.188
**No**	367 (65.7)	90 (61.2)	277 (67.2)	
**Yes**	192 (34.3)	57 (38.8)	135 (32.8)	
**Type of pregnancy at risk**				
**Anemia**	102 (46.57)	34 (50)	68 (45.03)	
**Gestational diabetes**	45 (20.54)	16 (23.53)	29 (19.21)	
**High blood pressure**	28 (12.78)	6 (8.82)	22 (14.57)	
**Pre-eclampsia**	6 (2.73)	0	6 (3.97)	
**Others**	38 (17.5)	12 (17.64)	26 (17.21)	

^1^ N: Number, (%): Percentage, RAMED: Medical Assistance Scheme for the Economically Underprivileged, CNOPS: the National Provident Organizations Fund, and CNSS: National Social Security Authority.

**Table 3:** List of medicinal plants used by pregnant women in the province of Guelmim during pregnancy, childbirth and at postpartum.

Family and Scientific Name	Vernacular Name	Mode of Preparation	Time	Reason for Use	Mode of Administration	FRC ^1^	UV ^2^
Alliaceae							
*Allium cepa* L.	Onion	Raw	TR2 ^3^	Prevention of miscarriages Vaginal itching/pruritus Prevention and treatment of genital infections	Vaginal	0.003	0.008
*Allium sativum* L.	Garlic	Raw	TR1 ^4^, TR2, TR3 ^5^	Urinary burns Pain Uterine involution Cold/flu/cough	Oral	0.013	0.011
Anacardiaceae							
*Pistacia lentiscus* L.	Lentisk	Fumigation	TR1, TR2	Prevention and treatment of genital infections	Vaginal	0.002	0.003
Apiaceae							
*Foeniculum vulgare* Mill.	Fennel	Powder Raw Decoction Infusion	Childbirth TR1, TR2, TR3 Postpartum	Intestinal bloating Good fetal development Pain Gastric pain Ease childbirth (accelerate labor) Promote the production of breast milk Prevent vomiting	Oral	0.016	0.019
*Petroselinum crispum* (Mill.) Fuss	Parsley	Raw Decoction	Childbirth TR2, TR3 Postpartum	Promote the production of breast milk Prevention and treatment of genital infections	Oral Vaginal	0.004	0.005
*Ammodaucus leucotrichus* Coss. Durieu	Hairy cumin or woolly cumin	Infusion Raw Powder	Childbirth TR1, TR2, TR3	Intestinal bloating Urinary burns Pain Gastric pain Ease childbirth (accelerate labor) Indigestion Induce labor (induction) Prevention and treatment of genital infections Cold/flu/cough Stress/anxiety	Oral	0.042	0.027
*Carum carvi* L.	Caraway	Infusion	Throughout pregnancy Postpartum	Vaginal itching/pruritus Promote the production of breast milk	Oral Vaginal	0.002	0.005
*Cuminum cyminum* L.	Cumin	Infusion Raw Powder	Childbirth TR1, TR2, TR3	Intestinal bloating Heartburn (heartburn) Constipation Diarrhea Pain Gastric pain Prevent miscarriages Ease childbirth (accelerate labor) Promote the production of breast milk Stress/anxiety Vomiting	Oral	0.029	0.032
*Daucus carota* L.	Carrot	Fumigation Decoction	Childbirth	Ease childbirth (accelerate labor)	Vaginal Oral	0.001	0.003
*Pimpinella anisum* L.	Green anise	Maceration	Childbirth TR1	Anemia Intestinal bloating Good fetal development Constipation Gastric pain Ease childbirth (accelerate labor)	Oral	0.006	0.016
Asteraceae							
*Artemisia herba-alba* Asso	White mugwort	Infusion Decoction Fumigation Raw Maceration	Childbirth TR1, TR2, TR3 Postpartum	Intestinal bloating Heartburn (heartburn) Urinary burns Constipation Vaginal itching/pruritus Pain Gastric pain Prevent bleeding Prevent caesarean section Ease childbirth (accelerate labor) Promote the production of breast milk Induce labor (induction) Uterine involution Loss of appetite Prevention and treatment of gestational diabetes Prevention and treatment of genital infections Cold/flu/cough Episiotomy care Treat high blood pressure (toxemia of pregnancy) Vomiting	Vaginal Oral Nasal	0.195	0.059
*Artemisia absinthium* L.	Absinthe	Infusion Decoction	TR1, TR2, TR3 Postpartum	Pain Ease childbirth (accelerate labor) Stress/anxiety	Oral	0.014	0.008
*Atractylis gummifera* L.	Slime thistle	Powder	TR3	Pain	Oral	0.001	0.003
*Matricaria chamomilla* L.	Chamomile	Infusion Decoction	TR1	Pain Gastric pain Stress/anxiety	Oral	0.005	0.008
Brassicaceae							
*Lepidium sativum* L.	Garden cress	Infusion Maceration Raw Decoction	Childbirth TR1, TR2, TR3 Postpartum	Anemia Intestinal bloating Pain Prevent bleeding Ease childbirth (accelerate labor) Promote the production of breast milk Induce labor (induction) Uterine involution Loss of appetite Prevention and treatment of genital infections Weight gain Cold/flu/cough	Oral	0.067	0.035
Burseraceae							
*Commiphora myrrha* (Nees) Engl.	Myrrh	Fumigation	Childbirth TR3	Ease childbirth (accelerate labor)	Vaginal	0.002	0.003
Boswelliasp.	Frankincense	Fumigation	Childbirth	Ease childbirth (accelerate labor)	Vaginal	0.001	0.003
Chenopodiaceae							
*Dysphania ambrosioides* (L.) Mosyakin and Clemants	Anserine	Maceration Decoction Infusion Powder	TR2, TR3	Fever	Dermal Oral	0.005	0.003
Cupressaceae							
*Juniperus communis* L.	Juniper	Decoction	Postpartum	Uterine involution	Dermal Vaginal	0.006	0.003
Fabaceae							
*Trigonella foenum-graecum* L.	Fenugreek	Decoction Infusion Maceration Raw Powder	Childbirth TR1, TR2, TR3 Postpartum	Anemia Heartburn Pain Gastric pain Ease childbirth (accelerate labor) Promote the production of breast milk Induce labor (induction) Uterine involution Loss of appetite Prevention and treatment of gestational diabetes Prevention and treatment of genital infections Weight gain Cold/flu/cough Vomiting	Oral	0.081	0.037
*Vicia faba* L.	Bean	Decoction	TR1, TR2	Anemia Heartburn (heartburn)	Oral	0.002	0.005
*Cicer arietinum* L.	Chickpea	Decoction	TR1	Anemia Heartburn (heartburn)	Oral	0.002	0.005
*Lens culinaris* L.	Lentil	Raw	TR1	Anemia Loss of appetite	Oral	0.002	0.005
Iridaceae							
*Crocus sativus* L.	Safran	Infusion Decoction Raw	Childbirth TR2, TR3	Intestinal bloating Pain Ease childbirth (accelerate labor) Stress/anxiety	Oral	0.021	0.011
Lamiaceae							
*Lavandula angustifolia* Mill.	Lavender	Infusion Fumigation Decoction	Childbirth TR1, TR2, TR3	Good fetal development Urinary burns Pain Gastric pain Induce labor (induction) Uterine involution Prevention and treatment of genital infections Cold/flu/cough Episiotomy care	Oral Vaginal Nasal Rectal	0.022	0.024
*Rosmarinus officinalis* L.	Rosemary	Decoction Infusion	Childbirth Throughout pregnancy TR1, TR2, TR3	Pain Ease childbirth (accelerate labor) Induce labor (induction) Prevention and treatment of genital infections Cold/flu/cough Stress/anxiety	Oral Nasal	0.026	0.019
*Salvia officinalis* L.	Common sage	Decoction Infusion	TR1, TR3	Good fetal development Pain Hormonal balance	Oral	0.005	0.008
*Thymus maroccanus* Ball.	Thyme	Infusion Decoction Raw	Childbirth TR1, TR2, TR3 Postpartum	Anemia Angina Intestinal bloating Good fetal development Constipation Pain Gastric pain Avoid perineal tears Ease childbirth (accelerate labor) Indigestion Induce labor (induction) Prevention and treatment of genital infections Cold/flu/cough Stress/anxiety Treat high blood pressure (toxemia of pregnancy) Vomiting	Oral Nasal Vaginal	0.153	0.045
*Mentha pulegium* L.	Pennyroyal mint	Infusion	TR1, TR2	Cold/flu/cough	Oral	0.004	0.005
Lauraceae							
*Cinnamomum verum* J. Presl	Cinnamon	Powder Decoction Infusion Maceration	Childbirth TR1, TR2, TR3 Postpartum	Anemia Urinary burns Pain Prevent bleeding Ease childbirth (accelerate labor) Promote the production of breast milk Induce labor (induction) Sore throat Prevention and treatment of genital infections Cold/flu/cough	Oral	0.019	0.027
Linaceae							
*Linum usitatissimum* L.	Lin	Powder Infusion	Childbirth TR1, TR2, TR3 Postpartum	Anemia Good fetal development Pain Promote the production of breast milk Indigestion Loss of appetite	Oral	0.008	0.016
Myrtaceae							
*Syzygium aromaticum* (L.) Merr. and Perry	Clove	Maceration Infusion Decoction Raw	TR1, TR2, TR3 Throughout pregnancy	Good fetal development Pain Gingival bleeding Induce labor (induction) Sore throat Prevention and treatment of genital infections Cold/flu/cough	Oral Nasal	0.016	0.019
Oleaceae							
*Olea europaea* L.	Olive	Decoction Infusion Nature	Childbirth TR1, TR2, TR3	Anemia Intestinal bloating Constipation Vaginal itching/pruritus Pain Prevent perineal tears Ease childbirth (accelerate labor) Loss of appetite Cold/flu/cough Stretch marks	Oral Vaginal Dermal	0.040	0.027
Palmaceae							
*Phoenix dactylifera* L.	Date	Raw	TR1, TR2	Urinary burns Loss of appetite	Oral	0.002	0.005
Pedaliaceae							
*Sesamum indicum* L.	Sesame	Powder Decoction Raw	Childbirth TR1, TR2, TR3 Postpartum	Anemia Good fetal development Constipation Gastric pain Prevent bleeding Promote the production of breast milk Loss of appetite Weight gain	Oral	0.041	0.021
Poaceae							
*Pennisetum typhoides* (Burm.f.) Stapf. and C.E. Hubb.	Candle millet	Powder	TR1, TR2	Anemia Good fetal development Loss of appetite	Oral	0.003	0.008
Ranunculaceae							
*Nigella sativa* L.	Nigella	Powder Decoction	Childbirth TR1, TR3	Good fetal development Pain Ease childbirth (accelerate labor) Induce labor (induction) Weight gain Cold/flu/cough	Oral	0.011	0.016
Rhamnaceae							
*Ziziphus lotus* (L.) Lam.	Jujube	Infusion	TR1	Pain	Oral	0.001	0.003
Verbenaceae							
*Aloysia citriodora* Palau	Verbena	Infusion Decoction	Childbirth TR1, TR2, TR3 Postpartum	Good fetal development Constipation Pain Gastric pain Eliminate intestinal gas in infants Ease childbirth (accelerate labor) Insomnia Uterine involution Cold/flu/cough Stress/anxiety Treat high blood pressure (toxemia of pregnancy) Vertigo Vomiting	Oral	0.097	0.037
*Vitex agnus-castus* L.	Chaste berry	Decoction Infusion	Childbirth	Ease childbirth (accelerate labor)	Oral	0.006	0.003
Zingiberaceae							
*Aframomum melegueta* (Roscoe) K. Schum.	Maniguette	Infusion	Childbirth TR3	Ease childbirth (accelerate labor)	Oral	0.001	0.003
*Zingiber officinale* Roscoe	Ginger	Decoction Infusion	Childbirth TR1, TR3	Vaginal itching/pruritus Pain Induce labor (induction) Cold/flu/cough	Oral	0.006	0.011
Zygophyllaceae							
*Peganum harmala* L.	Harmel	Fumigation Decoction	Childbirth TR1, TR3 Throughout pregnancy	Pain Ease childbirth (accelerate labor) Induce labor (induction) Cold/flu/cough	Nasal Vaginal	0.011	0.011

^1^ FRC: relative frequency of citation; ^2^ UV: use value; ^3^ TR2: 2nd trimester; ^4^ TR1: 1st trimester; and ^5^ TR3: 3rd trimester.

**Table 4:** Distribution of medicinal plants according to their mode of administration.

Application	At Birth %	Postpartum %	TR1%	TR2%	TR3%	Total %
Dermal	0.20	0	0	1.82	0.40	2.43
Nasal	0.20	0	1.82	0.91	0.91	3.84
Oral	15.57	4.85	22.65	16.48	13.65	73.21
Vaginal	12.74	0.20	4.65	0.71	2.22	20.53
Total	28.72	5.06	29.12	19.92	17.19	100

**Table 5:** Distribution of medicinal plants according to their mode of preparation.

Mode of Preparation	At Birth %	Immediate Postpartum %	TR1%	TR2%	TR3%	Total %
Decoction	8.76	0.20	10.26	9.56	6.08	34.86
Fumigation	3.69	0	1.99	0.70	0.70	7.07
Infusion	10.66	4.18	8.37	1.99	6.08	31.27
Maceration	0.10	0.10	0.60	0.20	0.10	1.10
Raw	2.19	0.50	6.27	5.98	3.39	18.33
Unidentified	3.09	0	0	0.10	0.10	3.29
Powder	1.39	0	1.10	1.10	0.50	4.08
Total	29.88	4.98	28.59	19.62	16.93	100

**Table 6:** Top sources of herbal medicine recommendations.

Source of Information	At Birth %	Pregnancy %	Postpartum %	Total %
Family	19	23	4	46
Friends and neighbors	15	16	3	34
Herbalists	1	2	1	4
Internet	1	1	0	3
Health professional	2	3	1	6
TV media	1	3	1	6
Total	41	49	10	100
